# A Combination of Ursolic Acid and Oleanolic Acid Disrupts Bioenergetic Homeostasis in Prostate Cancer and Inhibits Cancer Growth by Cytotoxic Autophagy

**DOI:** 10.3390/ijms27198448

**Published:** 2026-09-22

**Authors:** Kunj Bihari Gupta, Kanza Mamouni, Georgios Kallifatidis, Truette L. Taylor, Rizwana Zaman, Siva S. Panda, Bal. L. Lokeshwar

**Affiliations:** 1 Georgia Cancer Center, Augusta University, Augusta, GA 30912, USA; kmamouni@augusta.edu (K.M.); truettaylor8@gmail.com (T.L.T.); rzaman@augusta.edu (R.Z.); 2 Department of Biochemistry and Molecular Biology, Medical College of Georgia, Augusta University, Augusta, GA 30912, USA; sipanda@augusta.edu; 3 Department of Biological Sciences, College of Science and Mathematics, Augusta University, Augusta, GA 30912, USA; gkallifatidis@augusta.edu; 4 Department of Chemistry & Biochemistry, College of Science and Mathematics, Augusta University, Augusta, GA 30912, USA

**Keywords:** pentacyclic triterpenoids, ursolic acid, oleanolic acid, castration-resistant prostate cancer (CRPC), tumor growth inhibition, combination antitumor therapy, reactive oxygen species, cytotoxic autophagy, energy metabolism

## Abstract

Oleanolic acid (OA) and ursolic acid (UA) are bioactive pentacyclic triterpenoids (PTTs) and potential therapeutics for cancers. If tested individually, their therapeutic efficacy is limited. The antitumor potential of OA, UA, and their combination was tested against castration-resistant prostate cancer cells (CRPCs) in vitro and in vivo. UA, OA, and UA + OA (1:1) inhibited the cell proliferation and clonogenic growth of CRPC cells. Compared to the potency of individual OA and UA on these cells, their potency was synergistically enhanced when tested as an OA + UA mix. Neither of these compounds inhibited the proliferation of normal prostate epithelial cells. Further, in a CRPC (22Rv1) tumor model, the gavage of UA and, more effectively, the OA + UA mix inhibited tumor growth ≥ 60%, without measurable systemic toxicity. Mechanistic studies showed that these PTTs increase cyto-plasmic and mitochondrial oxidative stress. Cytotoxicity was predominantly triggered by high levels of reactive oxygen species (ROS). It was abolished when cells were co-treated with N-acetylcysteine (NAC), an oxygen scavenger and precursor for glutathione synthesis. The PTTs caused cell cycle arrest at the G1/S boundary, reduced glucose utilization, reprogrammed mitochondrial bioenergetics, and cytotoxic autophagy. Further, these data showed that cytotoxic autophagy is a significant contributor to PTT-induced tumor inhibition and OA + UA, at a 1:1 mix, selectively targets bioenergetics in prostate cancer cells, leading to ROS-mediated cytotoxic autophagy and robust tumor inhibition without significant systemic toxicity.

## 1. Introduction

Advanced, metastatic prostate carcinomas, also known as castration-resistant prostate cancers (CRPCs), are lethal and have few effective therapies to eradicate them [1]. An estimated 333,870 new prostate cancer (PCa) cases and 36,320 deaths in the United States are expected in 2026 [2]. All malignant cancers, including PCa, have unique metabolic adaptations that enable them to survive and proliferate [3]. PCa cells utilize aerobic glycolysis (the Warburg effect), a less efficient ATP generation system, as well as mitochondrial oxidative phosphorylation, to meet their energy requirements [4]. During aerobic glycolysis, glucose is converted into pyruvate, which is not effectively transported to the oxygen-consuming mitochondria using mitochondrial pyruvate carrier 1 and 2 (MPC-1 and 2). Tumor cells have evolved additional mechanisms for survival and meet their energy requirement by digesting and recycling their own cytosolic proteins (autophagy). Since unregulated autophagy may disrupt cellular homeostasis, this process adversely affects tumor cell survival, leading to cytotoxic autophagy [5]. Therefore, disrupting the energy conversion mechanism in CRPC cells could potentially kill the tumor cells and prolong patients’ survival. Agents that induce rapid autophagy in “energy-hungry”, rapidly growing tumor cells should provide an effective alternative therapy for PCa either as an adjuvant or mainstream therapy.

Pentacyclic triterpenoid compounds (PTTs) such as oleanolic acid (OA) and ursolic acid (UA), among others, show anti-cancer activity in multiple cancers [6,7]. These two phytochemicals, OA and UA (chemical structure shown below in Figure 1), are structural isomers that exhibit potent antiproliferative activities in several disease models, including neoplasia, inflammatory diseases, and hypertension. OA and UA show a divergent mechanism of anticancer potency [8,9,10,11].

Studies using only one of these compounds in antitumor chemotherapy as an adjuvant or neoadjuvant for recurrent cancers have shown limited efficacy due to their relatively low potency, with a 50% inhibition dose up to >30 micromolar in vitro [12], low bioavailability, or rapid metabolic degradation and elimination (in vivo) [13,14]. However, only a few studies have reported whether a combined UA and OA treatment is more efficacious [8,9].

In the present study, we investigated the efficacy of OA and UA, individually and in various combinations, for their therapeutic potential to reduce or eliminate tumor cell proliferation and tumor growth in vivo in experimental models of CRPC. We also explored the effect of the combination treatment on glucose metabolism, oxygen consumption rate, ATP production, mitochondrial integrity, and other properties of PCa to identify potential mechanisms by which OA and UA induce cytotoxicity and anti-tumor activities. We report that a combined OA and UA treatment in PCa cells and in vivo tumor models administered by oral gavage shows higher cancer-cell-specific toxicity, significantly alters metabolic pathways, and demonstrates plausible therapeutic use in patients with advanced PCa.

## 2. Results

### 2.1. Cytotoxic Activity of OA and UA on Different PCa Cells

Antiproliferative and cytotoxic activities of OA and UA individually and in combinations were tested in human androgen-receptor-expressing (AR^+^) LNCaP, C4-2, and 22Rv1 cells, and in non-responsive (AR^−^) PC3 cells. Cell viability following drug treatment for 72 h was estimated using the 3-(4,5-dimethylthiazol-2-yl)-2,5-diphenyltetrazolium bromide (MTT) reduction assay. As shown in Figure 2A, both OA and UA individually caused weak (OA) to moderate toxicity (UA) on this panel of PCa cell lines. However, when cells were treated with a 1:1 combination of OA + UA, the viability of all PCa cells was significantly decreased in a dose-dependent manner. Further, OA and UA individually and in combination did not induce cytotoxicity in spontaneously immortalized, non-tumorigenic prostate epithelial progenitor cells, NHPrE1 cells [15] (Supplementary Figure S1A). The 50% growth inhibition dose (IC-50) values for all tested cells are shown in Table A1.

Following the determination of the cytotoxicity of OA and UA, we determined their potential pharmacological synergy, additivity, or antagonism in PCa cells based on the principle of the dose–effect relationship using the CompuSyn analysis program [16]. The CompuSyn analysis program calculates the combination index (CI) to characterize drug interactions, where CI < 1, =1, and >1 represent synergistic, additive, and antagonistic effects, respectively. As shown in Figure 2C, at ≥10 µM of UA and OA, the pharmacological effect was synergistic, and, below that concentration, it was additive or largely supra-additive (CI ≤ 1). The dose–effect and median–effect graphs are shown in the supplementary figure (Supplementary Figure S1B). These results showed the potential to increase the efficacy of UA and OA in a 1:1 combination and reduce the dose of either drug for treatment without affecting the efficacy.

The MTT-based colorimetric viability assay detects the metabolic activity of cells but is not accurate in distinguishing between the cytotoxic and cytostatic effects of the drugs. Clonogenic cell survival assays (colony-forming assays) provide a more precise lethality estimation [17,18]. The ability to form colonies of cells following treatment with several doses of OA, UA, and OA + UA for 72 h was evaluated for both 22RV1 and PC3 cells. As shown in Figure 2B, the number and size of colonies were significantly reduced in both 22Rv1 and PC3 cultures in a dose-dependent manner upon treatment with OA and UA compared with untreated cells (control). More significantly, the combination index calculation from the colony formation assay showed that the combination treatment synergistically suppresses the clonogenic growth of both 22Rv1 and PC3 cells to a greater extent than when they were treated with OA or UA individually.

### 2.2. OA and UA Induce Oxidative Stress in PCa Cells

Reactive oxygen species (ROS) produced in the cells following drug exposure may cause oxidative stress and mitochondrial dysfunction, leading to cellular damage and cell death. ROS levels in the PCa cells were measured using a cell-permeable fluorescent molecular probe, dichlorofluorescein diacetate (DCFHDA). Intracellular esterases convert DCFHDA (a non-fluorescent probe) to dichlorofluorescin (DCFH), which is trapped inside the cells (cytoplasm). Following oxidation with nascent ROS, DCFH is converted into a fluorescent compound, dichlorofluorescein (DCF), and its conversion is directly proportional to the production of ROS [19]. As shown in Figure 3A, the levels of total ROS increased in a dose-dependent manner when cells were exposed to UA, OA, and UA + OA for eight hours.

Mitochondria are the major producers of both ROS (mt-ROS) and their neutralization [20]. We measured the levels of mitochondria-specific ROS (mt-ROS) using MitoSOX, a fluorescent molecular probe [21]. The PC-3 and 22Rv1 cell cultures were incubated with several doses of the drugs for eight hours, and cells were further incubated with MitoSOX for 30 min. We measured the fluorescence intensity of the MitoSOX probe using a plate reader (Bio-Tek Instruments, (Winooski, VT, USA)) [22]. As shown in Figure 3B, the level of mt-ROS is much higher than in the control, and we identified a dose-dependent increase in mt-ROS. Further, we noted that mt-ROS was significantly higher in cells incubated with OA + UA compared with those treated with OA or UA individually.

### 2.3. The ROS Scavenger, N-acetylcysteine (NAC), Eliminated OA + UA-Induced Oxidative Stress and Cytotoxicity

NAC is an established ROS scavenger and a precursor of L-Cysteine, which is required for glutathione synthesis, the endogenous antioxidant. It inhibits the intracellular production of ROS by promoting glutathione synthesis which neutralizes ROS [23]. We treated both 22Rv1 and PC3 cells with and without NAC, along with various concentrations of OA, UA, and OA + UA (1:1). Although NAC itself is non-toxic to these cells, it abolished OA- and UA-induced increases in both total cellular and mitochondria-specific ROS (Figure 3A,B). In both cases, NAC nearly nullified the effect of UA- and OA-induced oxidative stress. We determined the critical role of increased ROS in cell viability and its neutralization using NAC. Interestingly, incubating cells with NAC and the triterpenes not only decreased intracellular ROS levels but also abolished the triterpene-induced cytotoxicity in these cells (Figure 3C). These results showed that their ROS-inducing activity primarily mediates the cytotoxic mechanism of OA, UA, and their combination. Further, OA and UA primarily exert their cytotoxicity by generating excess ROS, which affects both mitochondrial oxidative phosphorylation and ATP generation in these cells.

### 2.4. UA and OA Arrested Cells in G1 Phase and Blocked Cell-Cycle Progression

PCa cells exposed to OA and UA for 24 h showed abnormal cell cycle phase distributions (Figure 4A,B). Cell cycle progression was arrested at the G1 phase in cell populations exposed to OA and UA alone, and when combined. Cell-cycle arrest at the G1 phase due to drug exposure was further confirmed by measuring the levels of cell-cycle-phase-specific proteins. As shown in the Western blot in Figure 4C, the protein levels of cyclin-E1 and cyclin-D1, both markers of G1-phase arrest, were higher in cells treated with OA, UA, and OA + UA compared with untreated PCa cells. Increased levels of Cyclin D1 and Cyclin E1 accelerate and deregulate the G1-to-S phase transition, causing the premature entry into DNA replication and genetic instability [24]. The observed increase in Cyclin D1 is indicative of cell cycle arrest at the G1/S transition. The activity of Cyclin D1-CDK4/6 complexes is also controlled by CDK inhibitors such as p21 protein and by the phosphorylation state of the Rb protein [25,26]. Therefore, elevated Cyclin D1 alone may not be sufficient to delay or block cell cycle progression. We examined the status of other G1/S transition regulators, including p21 and Cyclin A2, since p21 is a well-established inhibitor of Cyclin D/CDK and Cyclin E/CDK activity and Cyclin A2 is required for S-phase progression. As shown in Figure 4C, compared with the untreated cells, cells treated with OA, UA, and OA + UA were found to have a significantly increased p21 expression, and a decreasing Cyclin A2 expression. Although the fractions of cells in G1 increased significantly following treatment with the PTTs, and there was a significant decrease in S-phase fractions, there was no mitotic arrest resulting in an increase in the G2/M phase.

### 2.5. UA/OA Treatment Results in Mitochondrial Metabolic Reprogramming

The finding that levels of total-ROS and mt-ROS increase upon OA, UA, and OA + UA treatment in PCa cells prompted us to evaluate whether it alters the mitochondrial metabolic programming. To detect any alteration in mitochondrial bioenergetic pathways, we measured the cellular oxygen consumption rate (OCR) in 22Rv1 cells exposed to OA, UA, and OA + UA with the Seahorse XF Cell Mito Stress assay kit [27]. The mitochondrial respiration rate and OCR were measured in treated cells at baseline and FCCP-stimulated conditions (Figure 5A). Compared to untreated cells, thee basal OCR level decreased in the PTT-treated cell population.. Furthermore, upon treatment, Maximal Respiration and Spare Respiration were also significantly reduced (Figure 5B).

We measured the glucose uptake in 22RV1 cells incubated with PTTs for 24 h followed by incubation with fluorescein-labeled glucose (FL-G) for 30 min. The fluorescence intensity of cells was read in a plate reader. The uptake of FL-G was significantly lower in either OA- or UA-treated cells, but the decrease in FL-G uptake was greater in cells treated with UA. Further, the FL-G uptake in cells treated with the OA + UA combination was comparable to that of the UA-only treated cells (Figure 5C).

Next, we measured the glycolysis rate in 22Rv1 cells without any treatment to determine the basal glycolysis, which was calculated by subtracting the contribution of mitochondrial CO_2_ to the extracellular acidification rate (ECAR) from the total proton efflux rate. The rates showed that cells treated with OA and UA had decreased basal glycolysis (Figure 5D). Furthermore, compensatory glycolysis was calculated by measuring the glycolytic rate after treatment with inhibitors of mitochondrial oxidative phosphorylation (e.g., Rotenone and Antimycin A). We found significantly reduced compensatory glycolysis in cells treated with the OA + UA combination (10 µM each) compared to controls (Figure 5D).

Oxidative phosphorylation (OXPHOS) in the mitochondria and glycolysis in the cytosol are the two metabolic pathways responsible for ATP production [28], and we measured the rate of ATP production from these two pathways. The results showed that 22Rv1 cells relied mostly on mitochondrial OXPHOS rather than glycolysis. The glycolytic ATP production rate was slightly altered in OA + UA-treated cells (Figure 5D). In contrast, the mitochondrial ATP production rate was significantly decreased in OA + UA-treated 22Rv1 cells (Figure 5D), confirming a decrease in the rate of total ATP synthesis upon treatment with OA + UA.

### 2.6. UA/OA and Combination Treatment Decreases the Utilization of Mitochondrial Fuels—Glucose, Glutamine, and Fatty Acids

The cellular energy produced by mitochondria comes from three fuel sources: glucose, glutamine, and fatty acids. These are utilized at different rates to regulate various biological functions, including energy production, cell proliferation, and differentiation. We used the Mitochondrial Fuel Flex test kit (Agilent Technologies, 1503 Stevens Creek Road, Santa Clara, CA, USA) to examine the preferred fuel utilization of 22Rv1 cells after OA and UA treatment. Glucose, glutamine, and long-chain fatty acid (palmitate) oxidation were assessed through the sequential injections of fuel pathway inhibitors such as UK5099, BPTES, and etomoxir, respectively (Figure 5E). Specifically, by inhibiting these fuel pathways, we can determine the dependency, capacity, and flexibility of the cells to oxidize these three mitochondrial fuels. Our results show that cells treated with OA, UA, and the OA + UA combination exhibited a significant increase in the dependency on glucose and fatty acids (Figure 5F). The glutamine capacity was significantly reduced in the UA-, OA-, and OA + UA-treated cells compared to un-treated cells (Figure 5F). Overall, our fuel preference analysis suggests that the UA/OA-mediated impairment of mitochondrial oxidative metabolism may be due to the increased basal dependency and inhibition of glutamine metabolic stress response pathways caused by exposure to the PTTs (UA/OA).

### 2.7. UA/OA Treatment Changes the Central Carbon Metabolites

We analyzed the metabolite data for pathway over-representation using Metabolite Set Enrichment Analysis (MSEA). This analysis resolves biologically significant patterns due to drug treatment. The heat map of metabolite levels between the treated and control groups showed significant alterations. Broadly, the metabolites involved in glycolysis increased and those involved in the TCA cycle decreased (Figure 6A). Interestingly, the data from MSEA were quite similar to the pathway analysis, as the majority of metabolites that were altered are involved in cellular bioenergetics, as these metabolites significantly impact the TCA cycle, pyruvate metabolism, and glycolysis (Figure 6C). The top four significantly increased metabolites are Adenosine Diphosphate Ribose (ADPR), Dihydroxy-acetone Phosphate (DHAP), Phosphoenolpyruvate (PEP), and 3-Phosphoglyceric acid (3-PG). In contrast, the top four significantly decreased metabolites are Citrate, Isocitrate, cis-Aconitase, and Sedoheptulose-7-phosphate (S7P). These are shown in the volcano plots (Figure 6B). The area under the curve (AUC) plots for each metabolite category are shown in Supplementary Figure S2A–D. Principal component analysis (PCA), variable importance in projection (VIP), and correlations between metabolites are shown in Supplementary Figure S2E–G, respectively.

To further confirm the treatment-induced alteration of these metabolic pathways, we analyzed the enzyme levels of the TCA cycle by qPCR. We found elevated mRNA levels of the glycolytic enzymes (e.g., Triose-Phosphate Isomerase, Phosphoglycerate Kinase, α-Enolase, and Pyruvate Kinase) after 12 h and 24 h of PTT treatment in 22Rv1 and PC3 cells. Meanwhile, the mRNA levels of enzymes involved in the TCA cycle (e.g., Citrate Synthase and Aconitase-2) decreased in both cell lines at both post-treatment times (Figure 6E for 12 h and Figure 6F for 24 h). These findings align with the metabolomic variable importance in projection (VIP), and correlations between metabolites are shown in Supplementary Figure S2E–G, respectively.

### 2.8. Increased Autophagy, but Not Apoptosis, Is a Potential Contributor to OA/UA-Induced Cell Death

The antineoplastic drugs that impact mitochondrial integrity are known to trigger apoptosis or autophagy, or both. Since we observed the significant lethality of OA/UA on PCa cells, we probed the mechanism of apoptotic cell death by measuring the cleaved poly-ADP-ribose polymerase (c-PARP) levels through immunoblotting. The cleavage of PARP by a caspase-dependent process is a hallmark of apoptosis. As shown in Figure 7A, we were unable to detect any elevated c-PARP in either of the PCa cell lines after treatment with OA/UA, while cell lysates from Staurosporine-treated cells (a positive inducer of apoptosis) showed c-PARP, thus confirming the absence of apoptosis in OA/UA-treated cells. For further validation, we show the absence of other apoptosis markers, e.g., Bcl-2, Bax, Apoptosis Inducing Factor (AIF), AKT, and pAKT (Ser-473), in the PTT-treated cells.

We next measured the levels of biomarkers associated with autophagy, such as autophagy-related (ATG) proteins and the fatty-acylated LC-3B (LC3-BII), an autophagosome membrane protein. As shown in Figure 7B, LC3-BII levels were significantly increased in both 22Rv1 and PC3 cells treated with OA, UA, or OA + UA for 24 h. The proportion of the LC3-BII form was also higher in cells treated with rapamycin, a positive inducer of autophagy. To further confirm PTT-induced autophagy, we analyzed cell lysates prepared from treated cells for other autophagy-related proteins (SQSTM1/P62, Beclin-1, pULK-1, ULK-1, ATG-5, ATG-7, and ATG-13) by immunoblotting. The higher levels of expression of these proteins (Figure 7C) compared with untreated cells strongly support excessive autophagy and suggest a strong association between excessive autophagy, energy depletion, and cytotoxicity induced by OA, UA, and OA + UA.

### 2.9. Treatment with PTT Caused Reduction in Tumor Growth in a CRPC Tumor Model 22RV1 In Vivo

We evaluated the potential antitumor activities of low doses of OA and UA in 8-week-old male athymic mice bearing subcutaneously xenografted 22RV1 cells. Oral gavage with OA (50 mg/kg), UA (50 mg/kg), and OA (50 mg/kg) + UA (50 mg/kg) was initiated in groups of five mice each when the tumors were palpable (volume ≥ 3 mm^3^). Drug treatment was given through oral gavage, three times a week for six weeks or until the tumor volume of the vehicle-only group reached about 1000 mm^3^.

As shown in Figure 8A, the body weight of the tumor-bearing mice in the vehicle-only group or within treatment groups did not change significantly during treatment, indicating a lack of any adverse reaction to the drug treatment. Moreover, treatment did not induce any visible stress in mice or toxicity. These included lack of malaise, loose stools, or skin irritation (lesion), and bite-marks. These indicate that the PTTs given via avages were well-tolerated in mice. Tumor volumes were recorded as a measure of tumor growth throughout the treatment period and until the euthanasia of mice treated with OA, UA, and OA + UA. As shown in Figure 8B, compared to tumors in the solvent/vehicle only group the average tumor volume was significantly reduced in all treatment groups (Figure 8B). The tumor weights were compared for each group with the vehicle-only (control) group. Terminal tumor weights were significantly decreased in all treatment groups, when compared to the control (Figure 8C). Further, as shown in Supplementary Figure S3, based on the combination index (CI) calculation, there was a synergistic decrease in the tumor weights in the OA + UA group when compared to the tumor weights of the only-OA- or -UA-treated group. The tumors of each group at the time of euthanasia are shown in Figure 8D.

### 2.10. Increased Autophagy-Marker Levels in Tumor Tissues of Treated Groups

The formalin-fixed, paraffin-embedded tumor tissues were sectioned to perform histology and immunohistochemistry assays. Representative H&E-stained tumor sections for each group showed fewer nonuniform nuclei with distorted membranes and abundant vacuoles in the treated groups than in the vehicle-only gavage group, supporting the efficacy of UA and OA in reducing tumor growth (Figure 8E). Tissue sections were stained with anti-Ki-67 antibody to estimate cell proliferation activity. The density of nuclei that stained positive for the Ki-67 antigen was low in OA-, UA-, and OA + UA-treated tumor tissues compared with tissue sections in tumors from control animals. The tumor tissue sections were stained separately with Beclin-1 and SQSTM1/P62 (known markers for autophagic cell death) and demonstrate that the expression of both proteins was significantly higher in the treated tissue samples as compared to the control samples. This finding further confirms that, upon treatment with OA/UA, cytotoxic autophagy is the likely cause of the inhibition of tumor growth.

## 3. Discussion

Following a diagnosis of biochemical recurrence (post-surgery, rising PSA), men previously treated with surgery or radiation are now treated with hormonal therapy [29,30]. Predominantly, hormone therapy involves blocking growth signaling by either androgen ligand deprivation or antagonists of ligand-independent activities of the androgen receptor [31]. However, such treatments eventually fail, and the patients develop a resistance to antagonists of androgen and androgen receptors and, later, to cytotoxic chemotherapy. Palliative therapies are currently used to prolong patients’ lives but usually are not successful in stemming tumor growth beyond a short-term respite [32,33]. Additional therapies, including immunotherapy alone or adjuvant treatment regimens, are often used for such patients [34,35]. An approach that seeks to target cellular signaling mechanisms that do not directly affect cell replication, but, rather, cell death mechanisms, is often considered for treating advanced cancers either as monotherapies or adjuvant regimens (e.g., anti-angiogenic drugs). Our goal in this work was to utilize such mechanisms.

The potential of UA and OA as anticancer compounds has been extensively reported for several cancers, including breast and urogenital cancers [36,37,38,39]. These reports show that UA causes cytotoxicity via apoptosis or a combination of both apoptosis and autophagy in tumor cells, such as breast and colon cancer cells [12,40]. The mechanism by which UA exerts its antiproliferation activity is multifaceted, including the disruption of cellular metabolism, cell cycle alteration, and the inhibition of survival pathways. UA has been shown to increase the phosphorylation of BCL2 and the activation of caspase-3 and caspase-9 [39,41,42]. A group recently reported that UA interacts with all aspects of tumor physiology that are considered “hallmarks of cancer” [43,44]. However, few studies have compared the efficacy of the combined treatment with OA + UA with either agent alone. The data presented in this report confirms and extends the previously observed anti-cancer mechanisms of OA and UA. We found that, both individually and in combination, OA and UA block cell cycle progression, reduce clonogenic growth, and promote metabolic reprogramming. These activities likely led to cell death by the excessive generation of free-radical induced-mitochondrial dysfunction.

Unexpectedly, we observed the potent anti-proliferative activity of OA, UA, and OA + UA at lower concentrations (≤10 µM), compared to that reported in the past [43]. In terms of the range of published archives on the antiproliferative activities of UA, authors reported IC50 for UA > 30 µM. This value is significantly higher than the reported maximum plasma concentration (Cmax, ≤2.3 µM) in pharmacokinetics studies [45]. The median pharmacological concentration in plasma is shown in [46]. Thus, in our opinion, these studies may have a limited in vivo application of UA by the oral route. However, Shanmugam et al. reported, from their long-term study of a transgenic model of the prostate cancer, the transgenic adenocarcinoma of the mouse prostate (TRAMP), that UA in the solid feed showed a significant decrease in tumor formation and chemo-preventive efficacy in that model [36]. The tumor incidence and growth rate is significantly slower (~26-week) in TRAMP mice as compared to transplanted tumors. Therefore, the drug levels required for chemoprevention could be significantly lower than those required to inhibit established tumor cells. The lower potency of UA in cell-culture-based tests could be attributed to its ability to bind albumin in the serum present in the cell culture media. It has been reported that UA binds to bovine and human serum albumin, thereby reducing its uptake in vivo and tissue availability [47].

Other studies have shown that UA alone can induce apoptosis in cancer cell lines in vitro, at concentrations > 30 µM [47]. We noted UA to be cytotoxic to cells at <10 µM, but no detectable apoptosis was observed in cells treated with OA, UA, and OA + UA at concentrations (<20 µM) up to 72 h (Figure 7A). A plausible mechanism by which the OA + UA combination is more effective could be that the fraction of UA bound to albumin is lowered by competition with OA either in the cell culture (in vitro) or in vivo. In this scenario, the increased unbound UA interacts with its target and is effective at lower overall levels.

We show that the bioactive PTTs, OA and UA, have cytotoxic activity against PCa cells but not normal NHPrE1 cells; specifically, the two drugs show synergism when used in a 1:1 combination. Based on our cell cytotoxicity and clonogenic assay (Figure 2), we found that OA and UA are equally potent against cells that depend on androgens (LNCaP) and those that are androgen-depletion-resistant PCa (e.g., PC3) indicating that the combination may be potentially effective on a broad range of PCa subtypes, but, on the other hand, they are non-cytotoxic to non-tumorigenic prostate epithelial cells, NHPrE-1 (Supplementary Figure S1). Another aspect of using OA and UA is that they do not share similar pathways of cell death as traditional chemotherapy drugs [10,48].

We provide compelling evidence to show that PTTs induced cytotoxicity in CRPC cells by cytotoxic autophagy, probably due to cellular energy depletion and ROS-induced mitochondrial stress (Figure 3 and Figure 7C). Autophagy, the process by which tumor cells supplement their energy needs by recycling degraded organelles, protein aggregates, and other cytoplasmic components, may also function as a barrier to the treatment that targets cellular metabolism. However, autophagy could also be a “double-edged” weapon. The excessive stimulation of autophagy could result in tumor cells being unable to control the degradation of cellular components, leading to energy exhaustion and cell death.

We observed that excessive ROS induction in cells is a molecular mechanism of PTT cytotoxicity in tumor cells (Figure 3), as the inhibition of ROS by a scavenger, NAC, abolished the cytotoxicity of UA and OA. Although ROS are produced at a moderate rate during normal respiration and function as secondary messengers, regulate vasodilation, and kill pathogens, among other functions [49], excess and persistent ROS production induces oxidative stress, which adversely affects cell metabolism and macromolecular integrity, cumulatively causing cell death. Mitochondria, the bioenergetic center of the cell, are the most sensitive organelle that is affected by excess ROS. UA-induced excess ROS generation has been reported before [50]; however, its consequences for cell viability were not discussed, as in the present study. As shown in Figure 3C, we found that the cytotoxicity of UA, OA, and UA + OA can be significantly abolished by adding the ROS scavenger NAC. NAC did not affect the viability of untreated cells, whereas it reversed the cytotoxicity of the PTTs, which was more noticeable in the combination-treated cells. As shown in Figure 7B,C, CRPC cells treated with UA, and, to a lesser extent with OA, showed increased levels of typical autophagy markers such as LC3BII, Beclin-1, ATG-5, ATG-7, and ATG-13, all involved in autophagy progression during PTT-induced cell death. The levels of these markers in OA + UA-treated cells were significantly higher than indicated by their intensity in the blots.

Our in-depth analysis of oxygen consumption, glucose uptake, and ATP production in cells treated with PTTs at two concentrations showed a significant effect on CRPCs (Figure 5). The PTTs suppressed cellular oxygen and glucose uptake, reduced respiration, and increased oxidative glycolysis (Figure 5C). However, there was no significant decrease in proton leak (Figure 5B-bottom panel). This indicates the selective inhibition of ATP-linked respiration (coupled respiration) and the potential inhibition of ATP synthase. This metabolic profile in cells treated with UA or OA + UA may be attributed to the ability of these PTTs to specifically impair ATP generation through oxidative phosphorylation without damaging the structural integrity of the inner mitochondrial membrane. The lack of damage to mitochondrial integrity may explain why we did not observe the apoptotic activity of UA and OA at lower concentrations compared to what others have previously reported. Potentially, at higher concentrations, mitochondrial integrity is diminished, leading to cytochrome-c leak and caspase activation pathways leading to apoptotic cell death.

Our data showed cell cycle arrest at the G1 phase in cells treated with OA, UA, and OA + UA, which led to a decrease in S-phase cells. This may result from the decreased availability of DNA synthesis precursors caused by the disruption of mitochondrial energy production. We noted that the proportion of nuclei in the G1 phase was significantly higher in all treatment groups, including OA + UA, as compared to cells treated with the solvent alone (control). The molecular markers of G1 arrest, Cyclin D1 and E1, were higher in both CRPC cells, PC-3 and 22RV1, confirming the finding from the cell cycle analysis. A further analysis of protein markers of the G1 and G1/S phase such as Cyclin A2 and p21 corroborated our observation for the accumulation of UA-treated cells in the G1/S transition (Figure 4C). There is a decrease in Cyclin A2, a promoter of CDK2 activation, instead of an increase in the G1 to S-phase transition alongside an increase in p21, an inhibitor of CDK, which clearly indicates G1-phase arrest in cells treated with OA, UA, and OA + UA.

We report the antitumor activity of OA, UA, and OA + UA in a 22Rv1 CRPC xenograft model (Figure 8A–D) in athymic mice. The observed antitumor activity after oral administration suggests the potential clinical application of a specific oral formulation of OA, UA, and their 1:1 combination in patients with CRPC. The antitumor activity shown here by the delayed tumor growth, reduced tumor volume progression, and lower terminal tumor weight at necropsy following seven weeks suggests that the OA + UA combination is highly effective without any systemic toxicity in the experimental system (Figure 8A–C). The mean tumor weight of mice treated with OA + UA was decreased by >60% compared with untreated (control) mice. A potential mechanism of tumor cell death in treated tumors was autophagy, as indicated by fluorescent micrographs of Beclin-labeled tumor sections (Figure 8E). Overall, these studies show not only the high efficacy of PTTs as antitumor agents that can be delivered via oral gavage but also the lack of systemic toxicity as broadly indicated by the steady weights of the animals after seven weeks of treatment (Figure 8A).

In summary, we report a novel observation, indicating the potential eventual application in patients of the OA and UA combination as either a neoadjuvant or adjuvant therapy for CRPC, with minimal adverse reactions at the systemic level, yet effective antitumor activity using a well-established pair of PTTs. The two compounds OA and UA can function synergistically as anticancer drugs with a novel mode of toxicity mediated by cytotoxic autophagy. The potential of these drugs may be optimized by adjusting the treatment dose and further examining the cytotoxic mechanisms.

## 4. Materials and Methods

### 4.1. Reagents and Chemicals

All reagents were purchased from laboratory suppliers based in the USA, and details on specialty chemicals and detection kits used in this study are listed below in Table 1.

### 4.2. Cell Culturing

The PC3, LNCaP, and 22Rv1 were obtained from the ATCC (Manassas, VA, USA). All these cells were routinely cultured in a Complete Medium [RPMI1640 (Corning; Manassas, VA, USA) basal medium plus 10% fetal bovine serum (FBS, Biowest; Bradenton, FL, USA) and 2 mg/L gentamycin sulfate (IBI scientific, Dubuque, IA, USA)] [19]. C4-2 cells (a gift from Dr. Daqing Wu; Clark Atlanta University, Atlanta, GA, USA) were routinely cultured in T-medium (Gibco, Grand Island, NY, USA) with 5% FBS. NHPrE-1 cells, a kind gift from Dr. Simon Hayward, Northshore University Hospital, Chicago, IL, USA, were cultured in [DMEM-F12 (Corning; Manassas, VA, USA) basal medium plus 5% FBS, 1X Insulin-Transferrin-Selenium (Gibco), 50 mg/L Bovine Pituitary Extract (Gibco), 10 ng/L Epidermal growth factor, and 10 mg/L gentamycin sulfate] [15]. All cells were maintained at 37 °C in a humidified incubator with 5% carbon dioxide. Cell lines were genetically authenticated by Cell Line Authentication Services from Lab Corp Inc. (Burlington, NC, USA) and periodically tested against mycoplasma contaminants using a kit (Lonza Inc., Greenwood, SC, USA).

### 4.3. Cell Viability and Survival Assessment

The viability of different PCa cells after treatment with or without variable concentration of UA, OA, and its combinations was estimated using the colorimetric MTT assay as follows. Cells were seeded at a density of 2000/well in 96-well plates and treated with drugs for 72 h followed by incubation with MTT solution (50 µg/mL) for 3 h. The insoluble formazan crystal formed due to MTT reduction by live cells was solubilized in DMSO and the optical density (O.D.) of the solubilized formazan was read in a spectrophotometer at 570 nm/650 nm. The percent viable cells were calculated after subtracting to untreated controls [51]. We calculated the combination index (CI) for determining additive or synergistic interaction of combined UA and OA using the CompuSyn program (ComboSyn Incorporated; version 1.0, Paramus, NJ, USA) [16]. In the clonogenic cell survival assay, colonies were fixed with methanol after drug treatment and subsequently stained with 0.1% crystal violet. The number of surviving colonies, having more than 16 cells, were counted manually [51,52].

### 4.4. Reactive Oxygen Species (ROS) Measurement

Total ROS generated after treatment with OA, UA, and combination with and without N-acetyl cysteine (NAC) were estimated using a cell membrane permeable molecular probe 2′,7′-Dichlorodihydrofluorescein diacetate (DCFDA). Briefly, cells were cultured in 96-well plates at a density of 5000 cells per well and overnight cultured cells were treated with various drug concentrations. After completion of treatment, cells were washed with PBS followed by incubation with DCFDA (10 μM) for 30 min at 37 °C. The unbound dye was removed, and cells were rewashed with PBS, and the fluorescence intensity of each well was recorded using a microplate reader (BioTek Synergy HTX Multimode Reader, Agilent, Santa Clara, CA, USA) at an excitation wavelength of 485/20 nm and emission wavelength of 528/20 nm. Data is represented as percentage change related to the control [53,54].

The mitochondrial ROS (mt-ROS) levels generated in cells were measured using the MitoSOX™ dye with a procedure suggested by the vendor (Thermo-Fisher Inc., Eugene, OR, USA). The fluorescence intensity of each well was recorded using a microplate reader (BioTek Synergy HTX, Agilent, Santa Clara, CA, USA), and the data were represented as a percentage change from the control [18].

### 4.5. Flow-Cytometry-Based Cell Cycle Phase Fractionation

The PCa cells were cultured in 6-well plates at a density of 2 × 10^5^ cells/well overnight followed by treatment with various concentrations of OA, UA, and their combinations for 24 h. Single-cell nuclei suspension of cultured cells were prepared by suspending the cells in a hypotonic solution (50 mg Na-citrate, 15 μL of IGEPAL CA-630, and 0.1 mg/mL propidium iodide (PI)). Nuclei suspension stained with PI were fractionated on an analytical flow cytometer to determine the percentage of cells at various cell cycle phases based on the relative DNA content. Data acquired on the NovoCyte Quanteon Flow Cytometer (Agilent, Santa Clara, CA, USA) were analyzed using NovoExpress software (version 1.6.1) [18].

### 4.6. Estimation of Central Carbon Metabolites

The Central Carbon Metabolites which consist of metabolites related to glycolysis, Pentose-Phosphate pathways, Kreb cycle, and other related cofactors were estimated using liquid chromatography–mass spectrometry (LC-MS) as discussed previously [55]. Briefly, the overnight cultured cells were treated with drugs for 24 h. After completion of treatment, cells were collected and washed twice with PBS. Approximately 2–3 million cells were used for estimation of metabolites at the research core facility at Augusta University, GA. The metabolite data obtained was subjected to pathway over-representation analysis (ORA) of Metabolite Set Enrichment Analysis (MSEA), using MetaboAnalyst 5.0 [56].

### 4.7. Gene Expression by qPCR

Total RNA from treated and control cells was isolated using Aurum™ Total RNA Mini Kit (Bio-Rad Laboratories, Hercules, CA, USA) and cDNA was synthesized using iScript™ cDNA Synthesis Kit (Bio-Rad Laboratories) as per manufacturer protocol. The qPCR was performed on Applied Biosystems QuantStudio 3 Real-Time PCR system using the Universal SYBR Green Fast qPCR mix (Bio-Rad Laboratories) as described before [57]. The primer set used in this study was designed using the Primer3 tool and procured from Integrated DNA Technologies, Inc. (Coralville, IA, USA), listed in the below Table 2:

### 4.8. Immunoblotting

Whole-cell lysates were prepared in RIPA buffer supplemented with Pierce protease and phosphatase inhibitor cocktail from both treated and untreated cells. Total protein content was quantified with the Pierce™ BCA Protein Assay Kit (Thermo Fisher Scientific, Rockford, IL, USA). Volumes of cell lysates corresponding to 20 μg total protein/sample were mixed with SDS-Gel Sample buffer. They were then denatured and reduced by boiling them in 0.1 M DTT. Denatured samples were fractionated by SDS-PAGE and blotted onto a PVDF membrane (Merck Millipore, Tullagreen, Ireland #IPVH00010). The blots were incubated with indicated primary and secondary antibodies (listed in the Table 3) and developed to visualize protein bands using an enhanced chemiluminescence detection kit. Blot images were captured using the Bio-Rad ChemiDoc™ Touch imaging system. The blots of β-actin in each sample were used as an internal loading control.

### 4.9. Glucose Uptake Assay

The level of glucose uptake in the cells after treatment with various concentrations of UA, OA, and UA + OA was measured using Glucose Uptake-GloTM Assay kit (Promega, Madison, WI, USA). Briefly, cells (15,000 /well) were seeded in a 96-well plate and overnight cultured cells were treated with selected dose of UA, OA, and UA + OA for 24 h. After completion of treatment, cells were washed with PBS, and we followed the manufacturer instructions and finally record the luminescence of each well using GloMax™ 96 microplate luminometer (Promega Corp. Madison, WI, USA) [58].

### 4.10. Metabolic Stress Studies; Seahorse Assay to Measure Oxygen Consumption Rate

#### 4.10.1. Mitostress Assay

Agilent Seahorse XF Cell Mito Stress Test Kit was used to measure key parameters of mitochondrial function by directly measuring the oxygen consumption rate (OCR) of 22Rv1 cells treated with control (DMF) and various concentrations of UA, OA, and UA + OA for 24 h in complete medium (RPMI + 10%FBS). We followed the manufacturer instructions to measure OCR. Briefly, cells were plated with 15,000 cells/well (in 180 µL) in the Agilent Seahorse Cell Culture 96-well plate and incubated overnight in a CO_2_ incubator at 37 °C. The following day, cells were washed and supplied with assay medium (XF Base medium, 10 mM glucose, 1 mM pyruvate, 2 mM glutamine, and pH 7.4) and run with the XF96e Analyzer (Agilent Technologies, Santa Clara, CA, USA). The Seahorse cartridge was humidified with sterile water overnight in a non-CO_2_ incubator at 37 °C and submerged in prewarmed XF-calibrant solution for 45–60 min. Oligomycin, FCCP, and Rotenone/antimycin A solutions were prepared according to the manufacturer’s recommendations and added to cartridge port A, B, or C for a final concentration of 1 μM, 2 μM, and 0.5 μM, respectively. Oxygen consumption rate (OCR) was measured normalized to total cellular protein estimated using Micro BCA Protein Assay Kit. Results were analyzed in the WAVE software (Version 2.4.3.3) and processed by the Seahorse XF Cell Mito Stress Test Generator (Agilent Technologies). The measurements were also used to calculate ATP production according to the instructions of the manufacturer. The decrease in oxygen consumption rate following inhibition of the ATP synthase with oligomycin reflects the basal mitochondrial ATP production [58,59].

#### 4.10.2. Glycolytic Rate Assay

The glycolytic rate in the 22Rv1 cells after treatment with control (DMF) and various concentrations of UA, OA, and UA + OA for 24 h were measured using the Agilent Seahorse XF Glycolytic Rate Assay Kit (Agilent Technologies) following the manufacturer’s instructions. Briefly, cells were seeded at a density of 15,000 cells/well (in 180 µL) in the Agilent Seahorse Cell Culture 96 well plate and incubated overnight in a CO_2_ incubator at 37 °C. After completion of treatment, cells were washed and supplied with assay medium (XF RPMI medium, 10 mM glucose, 1 mM pyruvate, 2 mM glutamine, and pH 7.4) and analyzed with the XF96e Analyzer (Agilent). The Seahorse cartridge was humidified with sterile water overnight in a non-CO_2_ incubator at 37 °C and then submerged in prewarmed XF-calibrant solution for 45–60 min. Rotenone/Antimycin A and 2-deoxy-glucose (2-DG) solutions were prepared per manufacturer’s recommendations and added to cartridge port A or B for a final concentration of 0.5 μM and 50 mM, respectively. OCR and extracellular acidification rate (ECAR) were measured, and the glycolytic proton efflux rate (PER) was calculated in the WAVE software and processed with the Seahorse XF Glycolytic Rate Assay Generator per the manufacturer’s instructions. The calculated rates account for the contribution of citric-acid-cycle-derived CO_2_ to extracellular acidification and reflect glycolysis. Measured data were normalized to total cellular protein quantified by Micro BCA Protein Assay Kit (Thermo Fisher Scientific, Rockford, IL, USA) [58,59].

#### 4.10.3. Mitochondrial Fuel Flux Assays

The Mitochondrial Fuel Flex test kit was used to measure the basal state of mitochondrial fuel oxidation in live 22Rv1 cells. Cells were cultured in the Agilent Seahorse Cell Culture 96-well plate with a density of 15,000 cells/well (in 180 µL) and incubated overnight in a CO_2_ incubator at 37 °C, followed by treating the cells. After completion of treatment, we follow the manufacturer instructions for this assay. This test determines the cellular dependency of three major metabolic substrates (pyruvate, fatty acids, and glutamine) for mitochondrial fuel. The stock solution of commercially available specific inhibitors UK-5099 (inhibitor of mitochondrial pyruvate carrier (MPC)/glucose oxidation pathway; Sigma #PZ0160), Etomoxir Sodium Salt hydrate (inhibitor of carnitine palmitoyl-transferase 1A long-chain fatty acid oxidation; Sigma #E1905), and BPTES (inhibitor of the glutaminase/glutamine oxidation pathway; Sigma #SML0601) were prepared and added to cartridge port A or B as per the manufacturer’s recommendations. Baseline OCR was monitored for 18 min followed by sequential inhibitor injections (i.e., “Treatment 1” or “Treatment 2” below) with OCR readings for an hour following each treatment. The inhibitor treatments and calculations are as follows:

% Dependency = (Baseline OCR-Target Inhibitor OCR)/(Baseline OCR-All Inhibitors OCR) × 100%(1)

% Capacity = (Baseline OCR-Other 2 Inhibitors OCR)/(Baseline OCR-All Inhibitors OCR) × 100%(2)

### 4.11. In Vivo Studies to Test Antitumor Activities

All experimental procedures on mice were initiated and conducted using a protocol approved by the Augusta University Institutional Animal Care and Use Committee (IACUC, Augusta University Protocol No. 2016-0803). A statistical power analysis determined a group size of five per treatment with 80% power and a small error coefficient (α = 0.05), assuming a 100% tumor take rate with the PCa cell line 22Rv1. FOXN1 athymic male mice (Crl: NU(NCr)-Foxn1nu) were purchased from Charles River Laboratories (Durham, NC, USA). 22Rv1 cells (1 × 10^6^) were mixed with Corning^®^ Matrigel^®^ Matrix (Cat. No. 354234) in a 1:1 ratio and injected subcutaneously into the right flanks of 6-week-old athymic mice. Animals were randomized to the following treatment groups: vehicle control, UA (50 mg/kg), OA (50 mg/kg), and UA + OA (UA 50 mg/kg and OA 50 mg/kg). Mice’s health was monitored, and treatment began when all animals developed palpable tumors. Animals were treated three times per week via oral gavage with drugs suspended in 2% aqueous 4-hydroxy methyl cellulose with 1% sucrose and 0.02% Tween-80 (vehicle) which was prepared fresh every time [18,51]. The potential toxicity of the drugs in the experimental groups was assessed by visual observation at the time of tumor measurement and by recording the body weight of all mice individually. Mice weight and tumors were measured three times per week using a handheld digital caliper, and tumor volume was calculated using the following formula: (width × length × height × 0.53). Once the tumors in the vehicle control group averaged ≥500 mm^3^, mice were euthanized, and the tumors were dissected and weighed. Tumor tissues were fixed in formalin and processed for histology.

### 4.12. Immunofluorescence Staining of Tumor Tissues

The markers for cellular proliferations (Ki-67) and occurrence of autophagy (Beclin-1 & SQSTM1/P62) were evaluated on the 22Rv1-derived subcutaneous tumors by immunofluorescence staining. Briefly, the paraffin-embedded tumor tissues were first deparaffinized and rehydrated, followed by antigen retrieval by boiling in 10 mM sodium citrate solution + 0.05% Tween-20 solution for 30 min. Tissue sections were blocked with 3% BSA, and 10% goat serum for 1 h, followed by incubation with anti-Ki-67, anti-Beclin-1, and anti-SQSTM1/P62 primary antibody separately. After washing with PBS, the sections were incubated with fluorophore-labelled secondary antibody (Alexa Fluor 488) and nuclei were counterstained with DAPI. Finally, the sections were observed under the fluorescence microscope (BZ-X710, Keyence Corporation of America, Itasca, IL, USA) [58,60].

### 4.13. Quantification and Statistical Analysis

All in vitro treatments and measurements were performed using a minimum triplicate sample, and all experiments were repeated at least three times. All statistical analyses were performed using GraphPad Prism (GraphPad Inc., San Diego, CA, USA, Version 10.2.2). Unless otherwise indicated, data are presented as mean ± SD for control and experimental samples. We used Student’s *t*-test or ANOVA for all statistical validations of observed data. Significance was considered achieved with *p* < 0.05. Other details if specific are described in the respective figure legends.

## Figures and Tables

**Figure 1:**
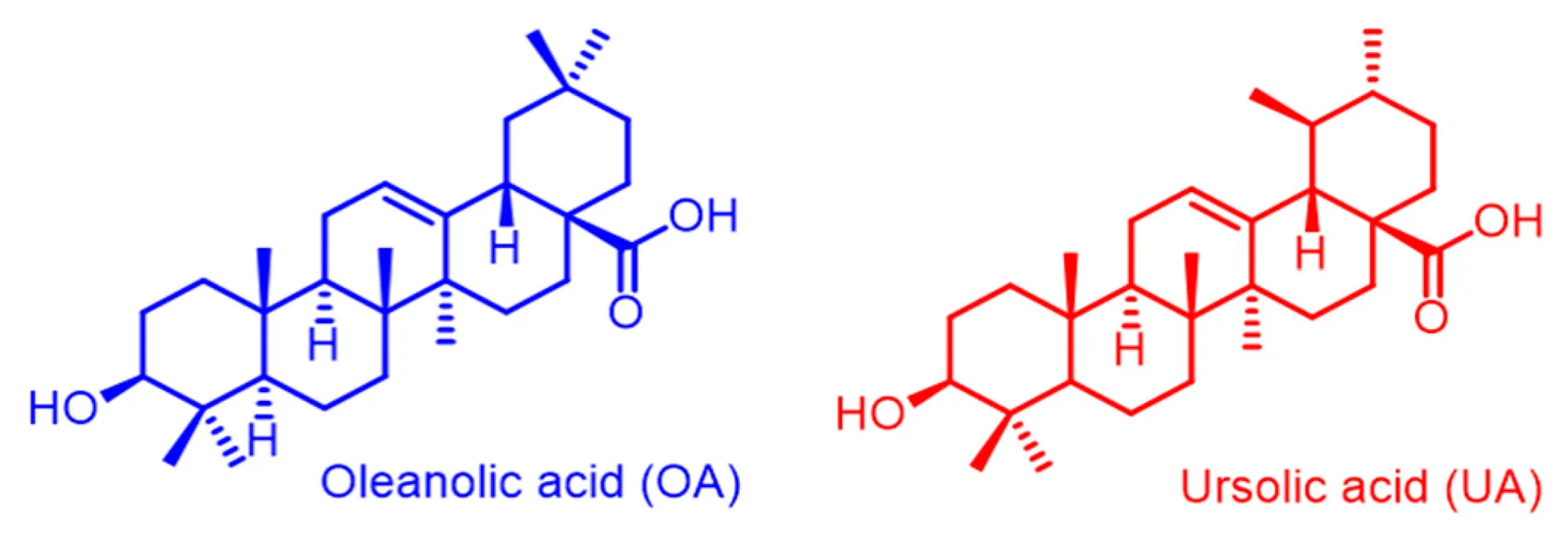
Chemical structure of OA and UA; both are structural isomers.

**Figure 2:**
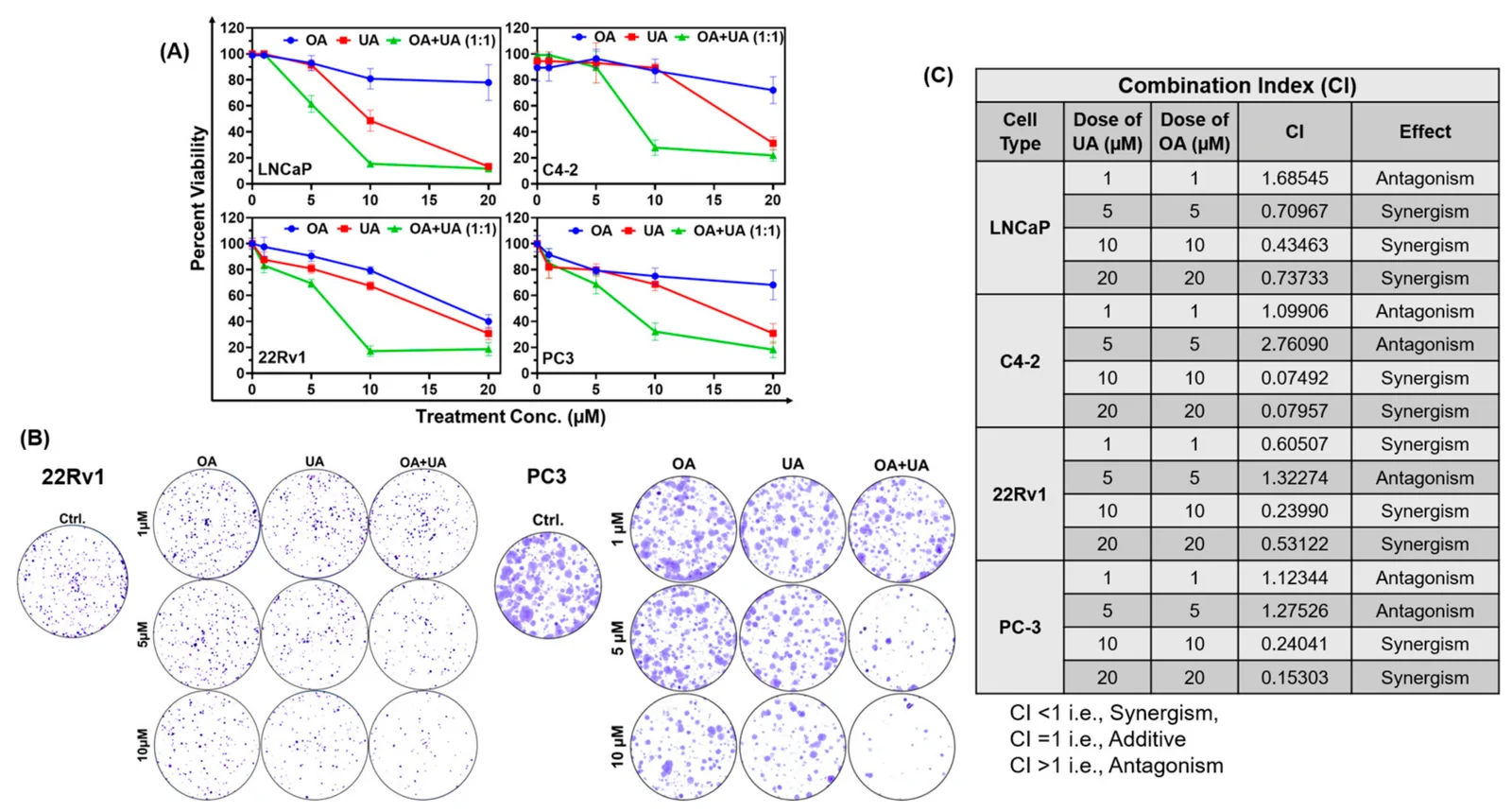
**Cytotoxicity of ursolic acid (UA) and oleanolic acid (OA) in PCa cells.** (**A**) Viability iability of several prostate cancer (PCa; LNCaP, C4-2, 22Rv1, and PC3) cell lines following treatment with UA, OA, and UA + OA (1:1) for 72 h. Cell viability was estimated using the MTT assay after 72 h of drug exposure. The percent viabilities were calculated with respect to the untreated cultures (control). Results were expressed as mean percentage viability ± Standard Deviation (SD) from three independent experiments, each with three replicates (n = 3). (**B**) Clonogenic survival (colony-formation) of 22Rv1 and PC3 cells was estimated by a colony formation assay after treating with UA, OA, and UA + OA for 72 h. Representative images are shown from three independent replicates (n = 3). (**C**) Combination index (CI) table for LNCaP, C4-2, 22Rv1, and PC3 cell lines. The CI was calculated using CompuSyn software version 1.0. CI was determined from dose–effect and isobologram analysis using optical density (OD) readings obtained from the colorimetry (MTT assay) data.

**Figure 3:**
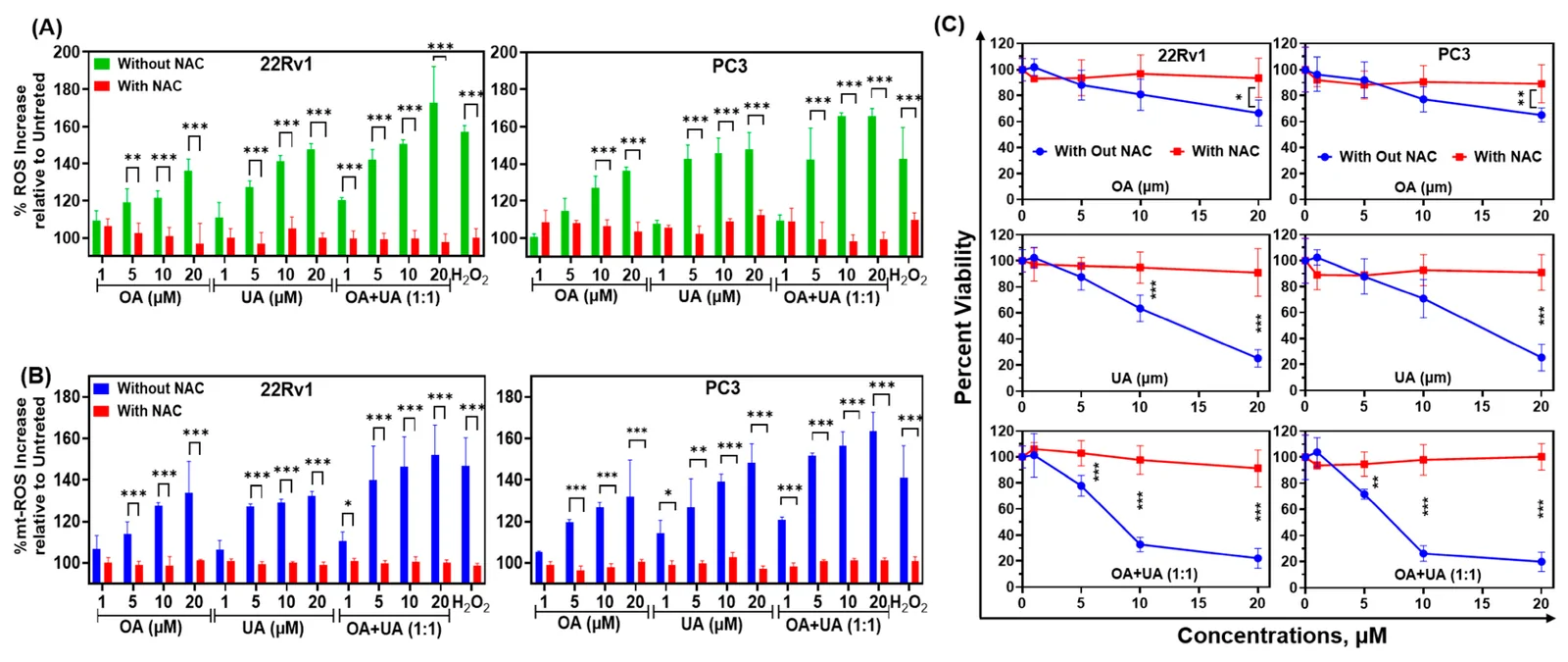
**OA and UA rapidly increase oxidative stress and induce cytotoxicity in PCa cells and N-acetylcysteine (NAC) abolishes these effects:** the production of total Reactive Oxygen Species (ROS) (**A**) and mitochondrial-specific ROS (mt-ROS) (**B**) in both 22Rv1 and PC3 cells after treatment with OA, UA, and OA+ UA (1:1), with and without NAC (5 mM). Cells treated with H_2_O_2_ (0.2 μM) served as positive control. The total ROS level was estimated using DCFDA, and mt-ROS was measured using MitoSOX detection kit (both are cell-membrane-permeable dye probes). (**C**) Percent cell viability was measured using MTT assay after treatment with OA, UA, and OA + UA with and without NAC. Data are presented as the mean ± SD (n = 4). Paired *t*-tests were used to determine the significance (*p*-value). * *p* ≤ 0.05,** *p* ≤ 0.01, *** *p* ≤ 0.005.

**Figure 4:**
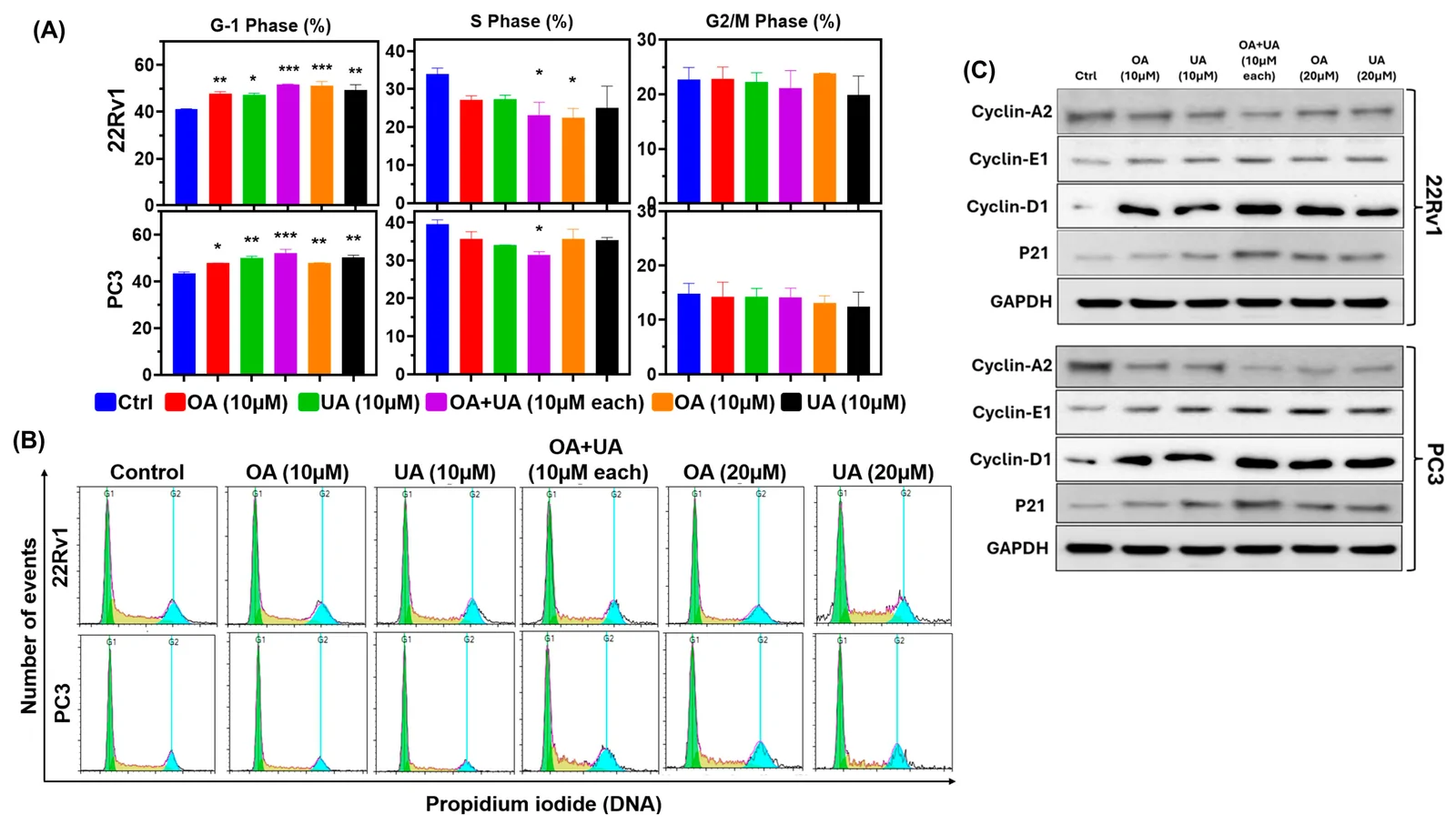
**UA- and OA-induced cytotoxicity via arresting cells in G1 phase.** Cell cycle phase resolution was analyzed using flowcytometry. Both 22Rv1 and PC3 cells were treated with OA, UA, and their combination for 24 h and stained with PI to determine cell cycle phase fractions. (**A**) The bar graphs showing the distribution of cell cycle phase fractions in each cell after treatment. (**B**) Representative cell distribution plots for each treatment in both cells. Bar colors indicate cell populations treated with control, specific drugs, or combination. (**C**) Immunoblots of Cyclin A2, Cyclin-E1, Cyclin-D1, and p21 in the total cell lysates after treatment, with GAPDH serving as an internal loading control. These blots were performed three times, and representative images are shown here. The percent of cells in each phase of the cell cycle are expressed as mean ± SD (n = 3). Paired *t*-tests were used to determine the significance (*p*-value). * *p* ≤ 0.05, ** *p* ≤ 0.01, *** *p* ≤ 0.005.

**Figure 5:**
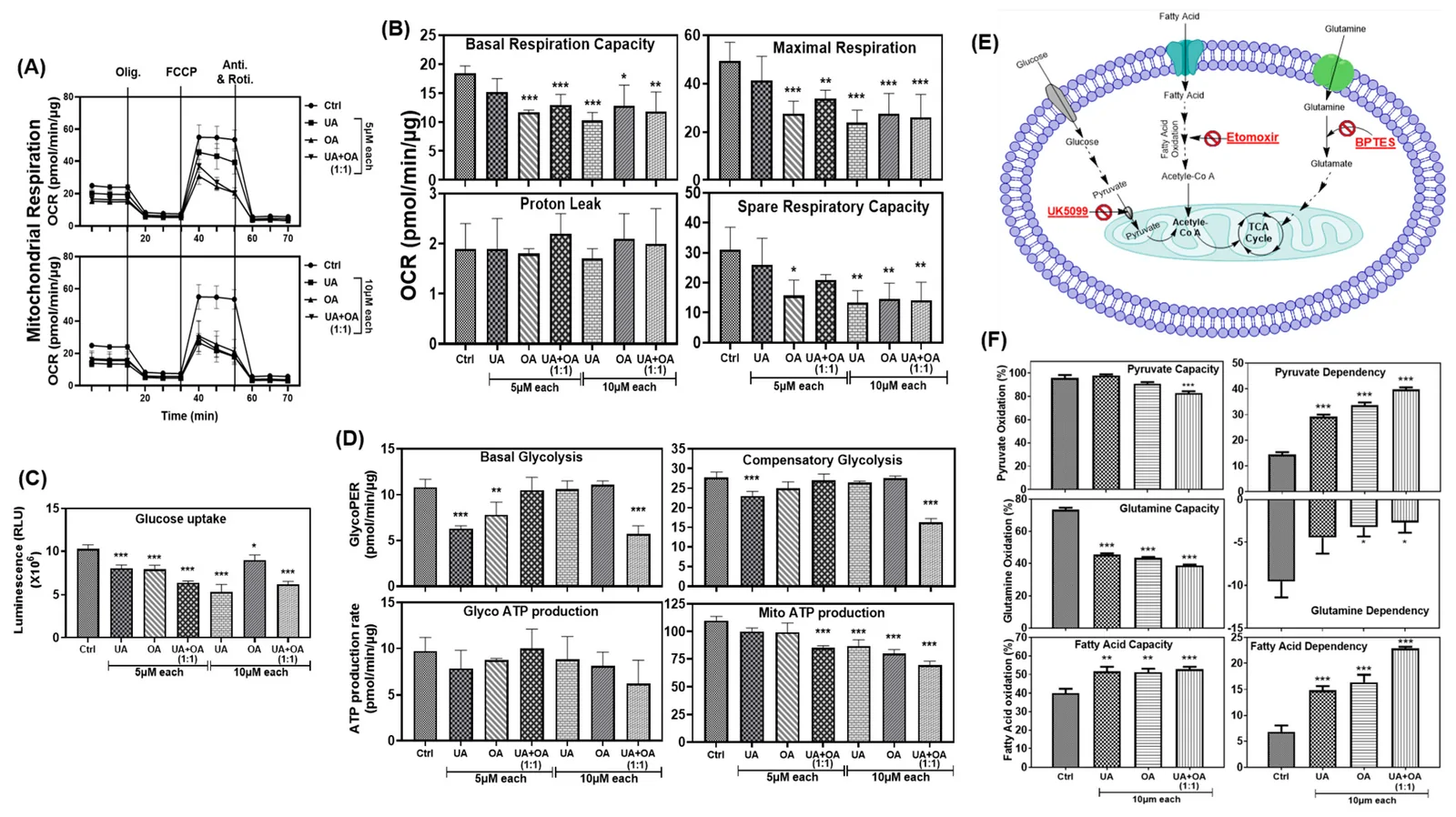
**Mitochondrial respiration function and fuel usage in 22Rv1 cells after treatment with OA, UA, and OA + UA.** (**A**) Oxygen consumption rate (OCR) changes after treatment with indicated concentrations of OA and UA for 24 h. (**B**) Bar charts showing the results of mitochondrial respiration changes analyzed with Basal Respiration, Maximal Respiration, Spare Respiratory Capacity, and proton leak for 24 h. (**C**) Total Glucose Uptake for 24 h. (**D**) Basal glycolysis proton efflux rate (Glyco PER), compensatory glycolysis (Glyco PER), calculated glycolytic ATP production rate, and calculated mitochondrial ATP production rate (per 10,000 cells) for 24 h. (**E**) Schematic representation of metabolites and different drug targets sites. (**F**) Percent capacity to use pyruvate, glutamine, and fatty acids for respiration was calculated by dividing OCR from pyruvate, glutamine, and fatty acids alone by the total mitochondrial OCR. Percent dependency on glutamine, pyruvate, or fatty acids was calculated as the decrease in oxygen consumption rate (OCR) after inhibiting that specific fuel pathway (expressed as a percentage of the total basal OCR). Data represented here are the mean ± SEM (n = 6). Significance was calculated using two-tailed unpaired *t* test (* *p* ≤ 0.05, ** *p* ≤ 0.01, *** *p* ≤ 0.005).

**Figure 6:**
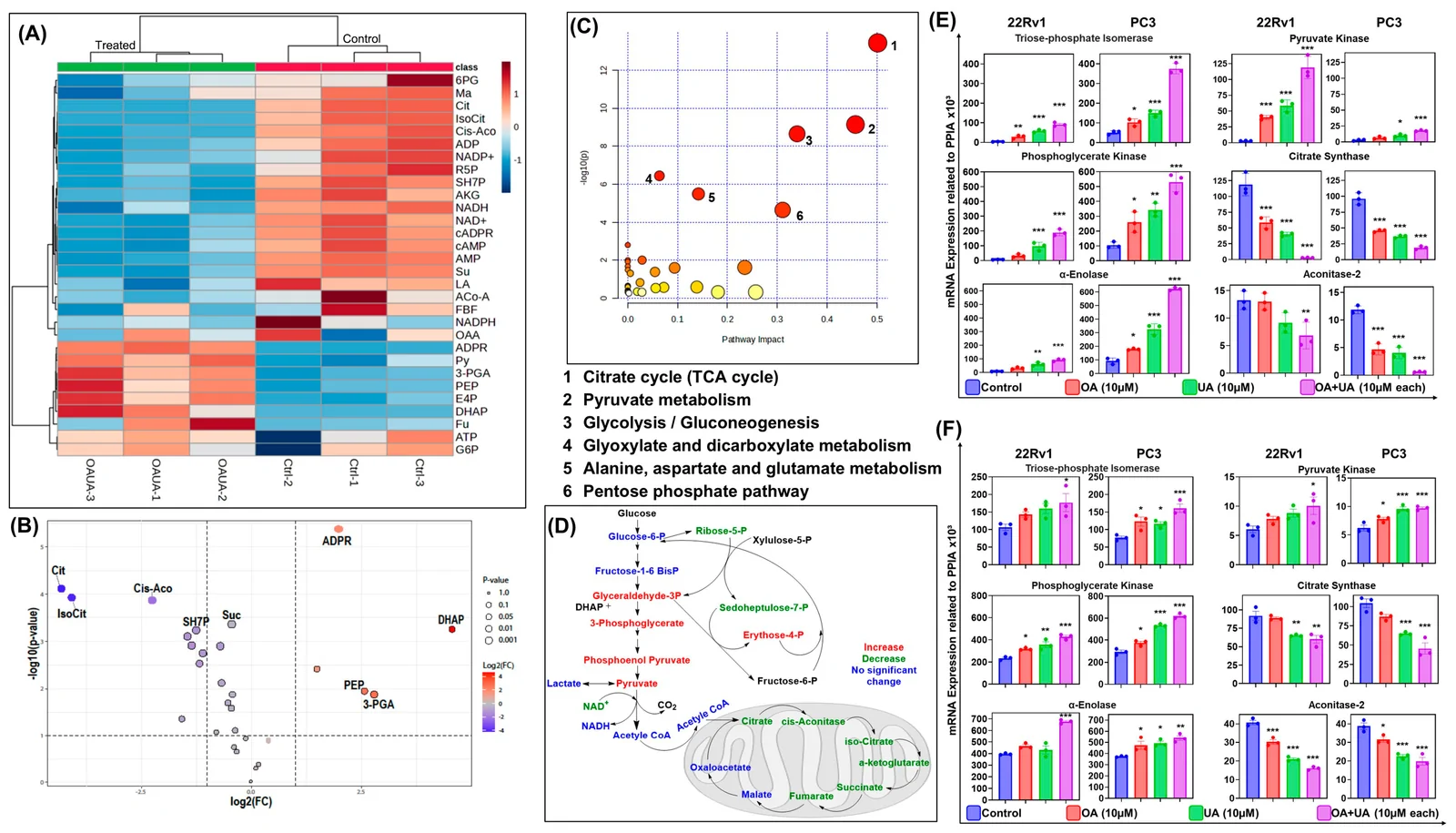
**OA and UA treatment significantly altered the level of Central Carbon Metabolites in 22Rv1 cells.** (**A**) Heat map showing the abundance of various metabolites between treated and control cells, after normalization. (**B**) Volcano-plot of all the metabolites in the linear model. The significantly positively enriched metabolites are shown in red, and the negatively enriched ones are represented by a blue gradient. (**C**) Joint-pathway analysis showing the impact of these metabolites on other pathways. We used heat map, volcano-plot, and pathway analysis using the Online software, MetaboAnalyst Version 5.0. (https://www.metaboanalyst.ca/). (**D**) Summary of treatment-induced metabolite alteration. The significantly upregulated, downregulated, and nonsignificant metabolites are depicted in red, green, and blue color, respectively. (**E**) After 12 h (**F**) and 24 h of treatment, qPCR-based gene expression analysis of genes involved in the central carbon metabolism, especially glycolysis and TCA cycle in both 22Rv1 and PC3 cell lines. The mRNA levels of all the tested genes were normalized to mRNA of peptidyl-prolyl isomerase A (PPIA) and final gene expression is shown as mean ± SD (n = 3). Paired *t*-tests were used to determine the significance (*p*-value). * *p* ≤ 0.05, ** *p* ≤ 0.01, *** *p* ≤ 0.005. Please see List of abbreviations at the end for the full name of the metabolites.

**Figure 7:**
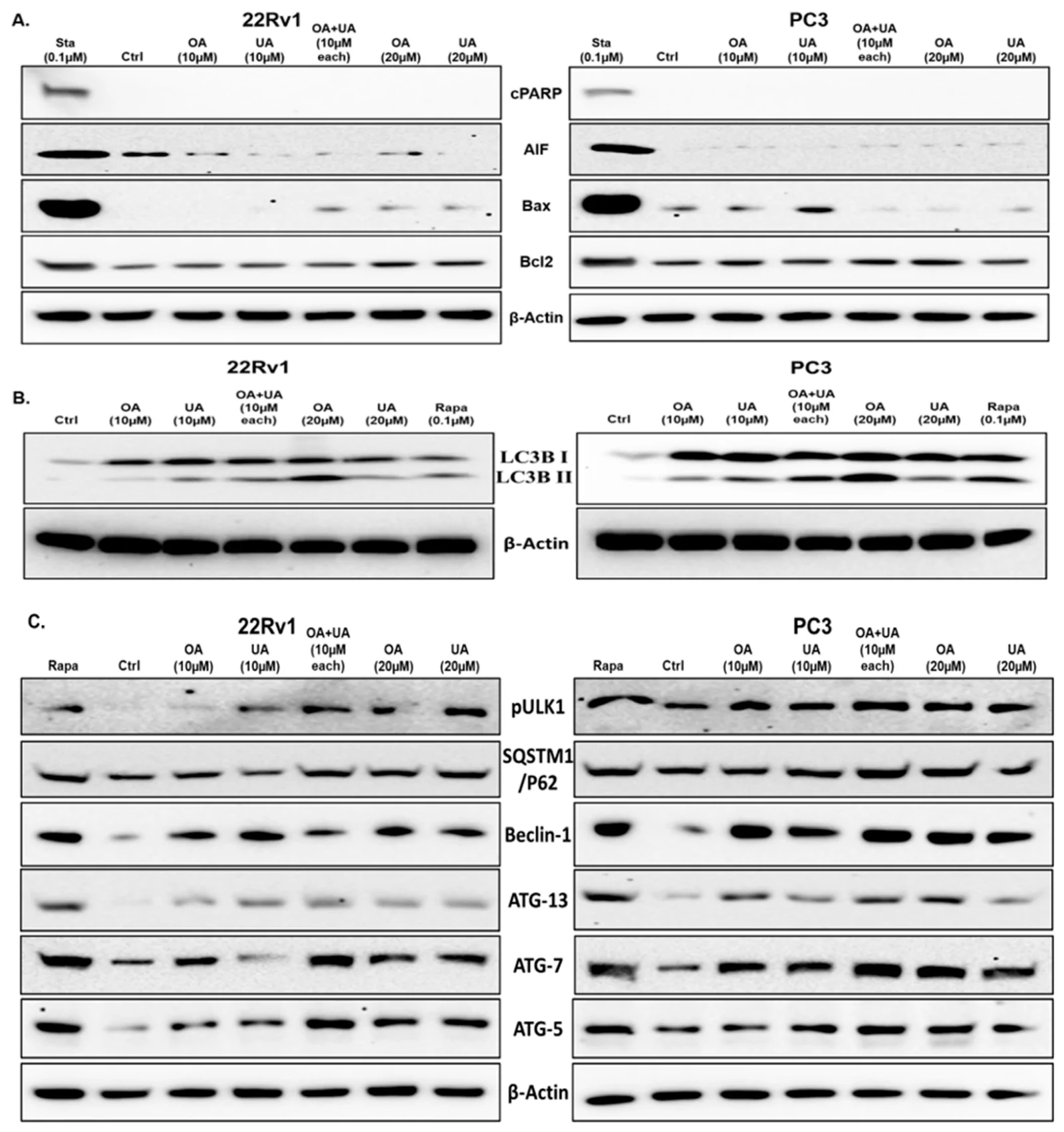
**Immunoblots of select-proteins involved in cell death mechanisms in 22Rv1 and PC3 cells after treatment with OA, UA, and OA + UA for 24 h:** (**A**) markers related to apoptotic cell death, (**B**) markers related to autophagic cell death, and (**C**) other associated proteins related to autophagy. Sta (Staurosporine: 0.1 µM) and Rapa (Rapamycin: 0.1 µM) are positive controls for apoptosis and autophagy induction, respectively. β-actin bands represent protein loading in each lane. The experiment was repeated three times; representative images are shown here.

**Figure 8:**
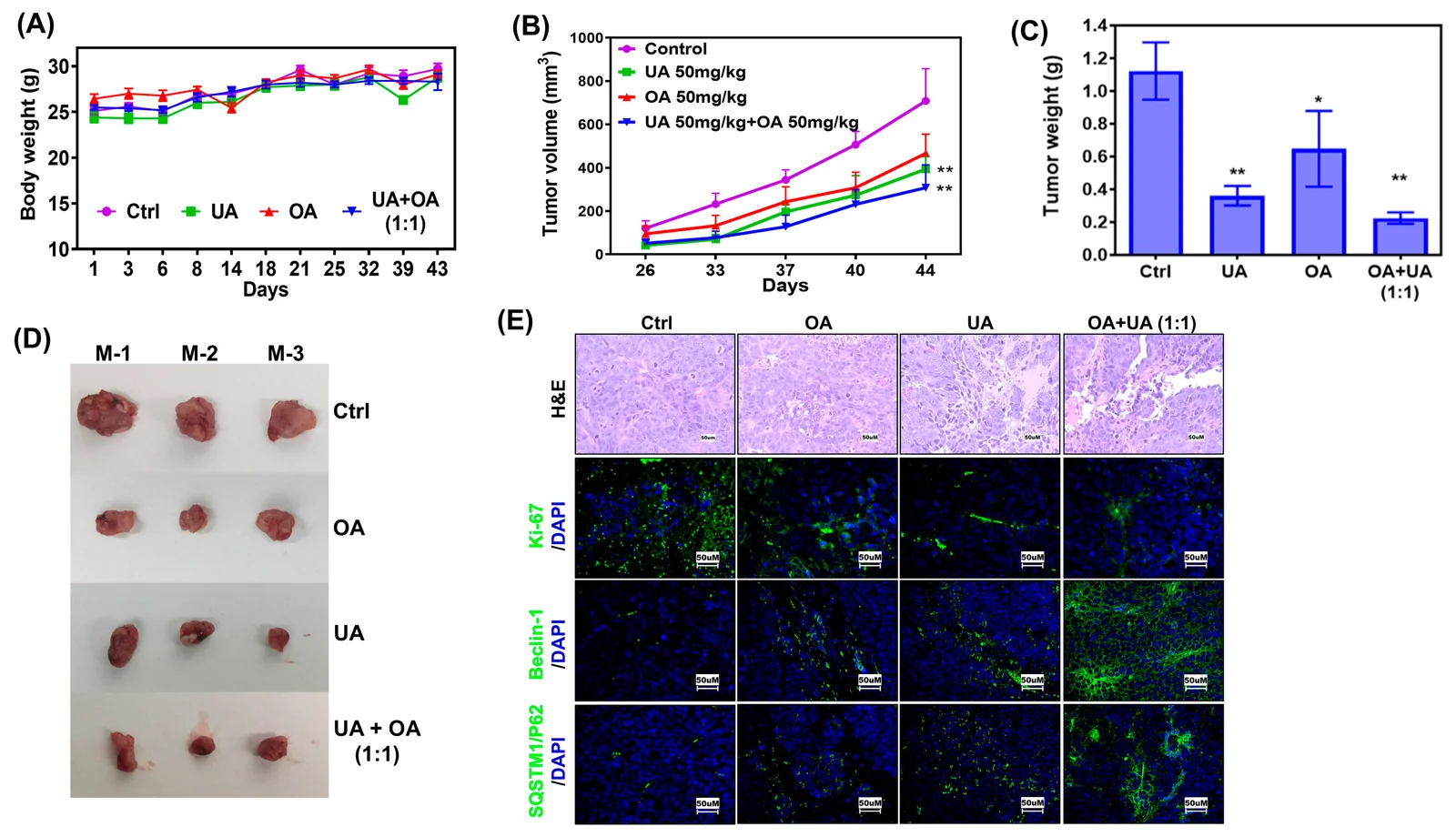
**In vivo effect of orally administered ursolic acid (UA; 50 mg/kg), oleanolic acid (OA; 50 mg/kg), and combination UA + OA (1:1; 50 mg/kg each) in athymic nude mice bearing 22Rv1 tumor:** (**A**) body weights of 22Rv1 tumor-bearing mice; (**B**) tumor volumes of 22Rv1 xenografts treated by oral gavage with vehicle control, UA, OA, and UA + OA (1:1); (**C**) weights of tumors at termination; (**D**) representative tumor images of each group; and (**E**) H&E-stained, Ki-67, Beclin-1, and SQSTM/P62 staining of tumor sections of each group to demonstrate the histological details of tumor cells, stroma, and activation of autophagy. Data are presented as mean ± SD, and paired *t*-tests were used to determine significance. * *p* ≤ 0.05, ** *p* ≤ 0.001. Bar scale 50 microns.

**Table 1:** List of fine chemicals and assay kits used in this study.

Fine Chemicals	Manufacturers with Catalog No.
Oleanolic acid (OA)	TCI America #O0317 (Portland, OR, USA)
Ursolic acid (UA)	Sigma-Aldrich #U6753 (St. Louis, MO, USA)
Rapamycin	LC Laboratories #R-5000 (Woburn, MA, USA)
Staurosporine	Fisher Scientific/MedChem Express #NC1401148 (Pittsburgh, PA, USA)
2′,7′-Dichlorodihydrofluorescein diacetate (DCFDA)	Sigma Aldrich #D6883
MitoSOX™	Thermo Fisher Scientific #M36008 (Eugene, OR, USA)
MTT	Alfa Aesar #L11939 (Ward Hill, MA, USA)
Propidium Iodide	Sigma Aldrich #P4170
N-acetyl cysteine (NAC)	Sigma Aldrich #A9165
Oligomycin	Sigma Aldrich
FCCP	Sigma Aldrich #C2920
Rotenone	Sigma Aldrich #R8875
Antimycin-A	Sigma Aldrich #A8674
2-deoxy-glucose	Sigma Aldrich #D8375
UK-5099	Sigma Aldrich #PZ0160
Etomoxir Sodium Salt hydrate	Sigma Aldrich #E1905
BPTES	Sigma Aldrich #SML0601

**Table 2:** List of gene and primer sequences used in the study.

Gene Name	Forward Primer (5′-3′)	Reverse Primer (5′-3′)
α-Enolase (ENO1)	GCAGGAATCCAGGTAGTGGG	CTCGGTCACGGAGCCAATC
Phosphoglycerate Kinase 1 (PGK1)	GACCTAATGTCCAAAGCTGAGAA	CAGCAGGTATGCCAGAAGCC
Cis-Aconitase 2 (ACO2)	GACTCGGCTGCAGAAAGCTC	CTTCTCCGAGAGTGTCAGCG
Triose-phosphate Isomerase (TPI)	AGTGACTAATGGGGCTTTTACTG	GCCCAATCAGCTCATCTGACTC
Pyruvate Kinase (PyK)	GAGAGCCACTATGGAACACCA	GGAGGTCTCAACACGAGGT
Citrate Synthase (CS)	TGCTTCCTCCACGAATTTGAAA	CCACCATACATCATGTCCACAG
Peptidylprolyl Isomerase A (PPIA)	TCTGCACTGCCAAGACTGAG	TCGAGTTGTCCACAGTCAGC

**Table 3:** List of antibodies used in this study.

Protein Name	Company with Cat. No.	RRID
Cyclin-E1	Epitomics #3327-1 (Burlingame, CA, USA)	AB_10640935
Cyclin-D1	Cell Signaling Technology #2978 (Danvers, MA, USA)	AB_2259616
AIF	Cell Signaling Technology #4642	AB_2224542
Bcl2	Proteintech #12789-1-AP (Chicago, IL, USA)	AB_2227948
Bax	Cell Signaling Technology #2772	AB_10695870
PARP	Cell Signaling Technology #9542	AB_2160739
pULK-1 (Ser757)	Cell Signaling Technology #6888	AB_10829226
P62/SQSTM1	Cell Signaling Technology #5114	AB_10624872
Beclin-1	Cell Signaling Technology #3738	AB_490837
ATG-5	Cell Signaling Technology #2630	AB_2062340
ATG-7	Cell Signaling Technology #2631	AB_2227783
ATG-13	Cell Signaling Technology #13273	AB_2798169
Cyclin A2	Cell Signaling Technology #67955	AB_2909603
P21	Cell Signaling Technology #2946	AB_2260325
GAPDH	Cell Signaling Technology #2218	AB_561053
LC3B	ABclonal #A7198 (Woburn, MA, USA)	AB_2863546
Ki-67	Abcam #AB16667 (Cambridge, UK)	AB_302459
β-Actin	Proteintech #HRP-60008	AB_2819183
Anti-mouse Secondary	Cell Signaling Technology #7076	AB_330924
Anti-rabbit Secondary	Cell Signaling Technology #7074	AB_2099233
Fluorescence-labeled Secondary Antibody	Goat anti-Rabbit IgG (H + L) Alexa Fluor™ 488 #A11008 (Invitrogen, Waltham, MA, USA)	AB_143165

## Data Availability

The original contributions presented in this study are included in the article/Supplementary Materials. Further inquiries can be directed to the corresponding author(s).

## Supplementary Materials

The following supporting information can be downloaded at: https://www.mdpi.com/article/10.3390/ijms27198448/s1.

## Abbreviations

The following abbreviations are used in this manuscript:

AR	Androgen receptor
CI	Combination index
CRPC	Castration-resistant prostate cancer
ECAR	Extracellular acidification rate
IC50	50% inhibition concentration
MSEA	Metabolite set enrichment analysis
NAC	N-Acetyl Cysteine
OA	Oleanolic acid
OCR	Oxygen consumption rate
PCa	Prostate cancer
PI	Propidium Iodide
PTT	Pentacyclic triterpenoid
ROS	Reactive oxygen species
UA	Ursolic acid
Py	Pyruvate
LA	Lactic Acid
Fu	Fumarate
Su	Succinate
OAA	Oxaloacetate
Ma	Malate
AKG	Alpha-ketoglutarate
PEP	Phospho Enrol Pyruvate
DHAP	Dihydroxyacetone phosphate
Cis-Aco	cis-Aconitate
IsoCit	Isocitrate
Cit	Citrate
E4P	Erythrose 4-phosphate
R5P	Ribose 5-phosphate
G6P	Glucose-6 phosphate
6PG	6-phosphogluconate
SH7P	Sedoheptulose 7-phosphate
cAMP	Cyclic Adenosine Monophosphate
FBF	Fructose-1,6-bisphosphatase
ACo-A	Acetyl-Coenzyme A
ADP	Adenosine diphosphate
ATP	Adenosine triphosphate
cADPR	Cyclic Adenosine diphosphate Ribose
ADPR	Adenosine Diphosphate Ribose
3-PGA	3-Phosphoglyceric Acid
AMP	Adenosine monophosphate
NAD+	Nicotinamide Adenine Dinucleotide+
NADP+	NAD Phosphate+
NADH	NAD Hydrogen
NADPH	NADP Hydrogen

## Appendix A

**Table A1:** IC-50 conc. (µM) of different drugs in different PCa cell lines.

Drug Name and Dose (µM)	Cell Type			
	22Rv1	PC3	LNCaP	C4-2
OA	17.01	>20	>20	>20
UA	13.44	13.62	9.97	16.07
UA + OA (1:1)	6.27	6.95	5.85	8.29
